# Validation of a novel clinical tool for monitoring distal limb stiffness

**DOI:** 10.3389/fvets.2023.1271036

**Published:** 2024-01-05

**Authors:** Benjamin D. Jacklin, Katherine Hanousek, Sabrina Gillespie, Anna Liedtke, Rachel Tucker, Andrew Fiske-Jackson, Roger K. Smith

**Affiliations:** ^1^CVS Group Plc, Suffolk, United Kingdom; ^2^Equine Referral Hospital, Royal Veterinary College, Hatfield, United Kingdom; ^3^Liphook Equine Hospital, Hampshire, United Kingdom

**Keywords:** tendon, tendinopathy, tendinitis, goniometer, racehorse

## Abstract

**Objective:**

To validate a novel technique to measure limb stiffness in a clinical setting.

**Animals:**

Three horses and three ponies owned by the Royal Veterinary College.

**Procedures:**

Limb stiffness indices for both forelimbs were first derived using the gold standard of kinematic analysis. Using the same animals, limb stiffness indices were then calculated using portable floor scales to record weight and an electrogoniometer to record changes in metacarpophalangeal joint angle. The two techniques were then assessed for correlation and repeatability.

**Results:**

The repeatability of limb stiffness measurement using the novel clinical tool was considered to be good based on a small coefficient of variation (5.70%). The correlation of limb stiffness as derived by both methods was high (*r* = 0.78, *p* < 0.01). Limb stiffness was positively correlated with the mass of the subject (*r* = 0.85, *p* < 0.01), with heavier horses having greater limb stiffness.

**Clinical relevance:**

This study has compared a novel method to measure distal forelimb stiffness non-invasively in a clinical setting to kinematic analysis in six equids. It has demonstrated that limb stiffness increases in a linear fashion with body mass consistent with the role of forelimbs providing energy storage. Because *in vivo* limb stiffness has been shown previously to alter with injury to the superficial digital flexor tendon, it is hypothesized that this technique will offer a practical technique for the clinician to assess limb stiffness in clinical cases. Further study will be necessary to determine its clinical usefulness in such cases.

## Introduction

Superficial digital flexor tendon (SDFT) tendinopathy is a common cause of wastage and enforced retirement among equine athletes (1–6). Racing thoroughbreds have been shown to be highly susceptible to SDFT tendinopathy, affecting approximately 24% of racehorses in training and up to 40% of animals on individual training yards (1).

The superficial digital flexor tendon plays a key role in storing energy on weight-bearing for efficient locomotion, and so its mechanical properties are optimized for this purpose (7). Acute clinical injuries are thought to be the end result of cumulative degeneration of the tendon extracellular matrix, as the tendon undergoes repetitive strain over time, with a reduced capacity for remodeling as the animal and tendon age (8–13). While persistent lameness is rarely an issue with SDFT tendinopathies, re-injury is common, with a reported prevalence of 23–67%, thought to be due to inferior biomechanical function of the healed tendon (6, 14, 15). Monitoring of clinical progress following injury is currently performed using ultrasound, which has been shown to have some correlation with histological findings in healing tendons (16, 17). However, this offers little information regarding the biomechanical competence of the SDFT as lesions heal, and decision-making with regard to increasing exercise planes can be challenging for the clinician.

A previous study demonstrated a close correlation between limb stiffness findings of horses, determined using motion capture and force plates *in vivo,* with the *in vitro* stiffness of SDF tendons at post-mortem (18). Unilaterally injured (SDFT tendonitis) limbs were found to have markedly reduced limb stiffness following injury, which increased over a 7-month period by which time it approximated that of the contralateral (normal) limb. This offers the intriguing possibility that measurement of the return to normal limb stiffness following injury may be used as a monitoring tool to guide rehabilitation. However, the techniques described in this study were complex and required high-value equipment and expertise in its use and as such are not suitable for use in the clinical setting.

Goniometry has been used and validated in human biomechanics and physiotherapy for accurate measurement of joint angles *in vivo* (19–22). Reliability and accuracy are improved when simple “hinge” joints are measured (23). Studies have also found goniometric measurements of joint angles to be reliable in cats, dogs, and horses (21, 24–26). The use of goniometers in horses has been described for the assessment of a passive range of motions in both standing and sedated animals (24, 27).

The objectives of the current study were 2-fold: (1) to compare the use of an electrogoniometer and floor scales with kinematic analysis as a technique for measuring limb stiffness in a clinical setting; and (2) to investigate the relationship between body mass and limb stiffness in the horse.

## Materials and methods

Six university-owned equids, 3 horses and 3 ponies (age range 6–24 years), were used in the study, which was approved by the ethics committee of the Royal Veterinary College (SSRERB number: 2013/R56). All were mixed native breeds and kept at pasture. Animals were unshod and subject to a 6-weekly foot trimming regimen by a registered farrier. All animals were evaluated to confirm an absence of lameness by subjective evaluation when trotting in a straight line and an absence of palpable abnormalities of the distal thoracic limbs prior to each stage of the study. This was performed by an experienced equine practitioner (BJ). The animals were weighed using equine weigh scales and weighed between 258 and 506 kg. Limb stiffness (as determined by the force (kg) needed to change the metacarpophalangeal angle by one degree (kg/degree)) was determined for both thoracic limbs in each animal, using both of two different methods as follows. Both methods were performed on the same day, with method 1 performed prior to method 2.

### Method 1: Kinematic determination of limb stiffness

Animals were evaluated in the manner described previously (18). Retroreflective markers were precisely positioned on both forelimbs on the skin overlying the head of the fourth metacarpal bones, the lateral epicondylar fossae of the third metacarpal bones, and the lateral hoof wall at the center of rotation of the distal interphalangeal joint (8). Repeatability and accuracy of these methods have been established in the equine distal limb (28, 29).

The positions of the retroreflective markers were recorded using eight Qualisys (Software: QTM V2.0.365/Hardware Oqus 3 Series) three-dimensional computerized motion analysis cameras (Kistler Instrumente AG, Winterthur, Switzerland) supported on individual tripods. These were positioned on either side of seven force plates (Kistler 9287BA) (Qualisys AB, Gothenburg, Sweden) laid in series, over which each animal was walked in a straight line to measure vertical ground reaction force (GRF). Horses were walked over the force plates and between the cameras until each thoracic limb had completed ten or more successful “strikes” on a single force plate. A successful strike was defined as a foot placement where the entire solar surface landed in the center of a single force plate and was withdrawn from the same before other limbs struck the same force plate.

The vertical GRF was not standardized per kilogram bodyweight in order to assess the influence of the mass of each animal on derived limb stiffness. Data were analyzed using commercial software (The MathWorks Ltd, Cambridge, United Kingdom). The derived metacarpophalangeal (MCP) joint angles and their associated vertical GRF data points were both standardized to 100 points evenly distributed across the stride phase, enabling comparison between strides. A force–deformation plot was thus generated for each stride, and the gradient of the linear “unloading” portion of the curve was determined. A mean of these derived gradients was taken from both thoracic limbs of the horses, generating a “limb stiffness index” for each.

### Method 2: Goniometric determination of limb stiffness

**Figure 1:**
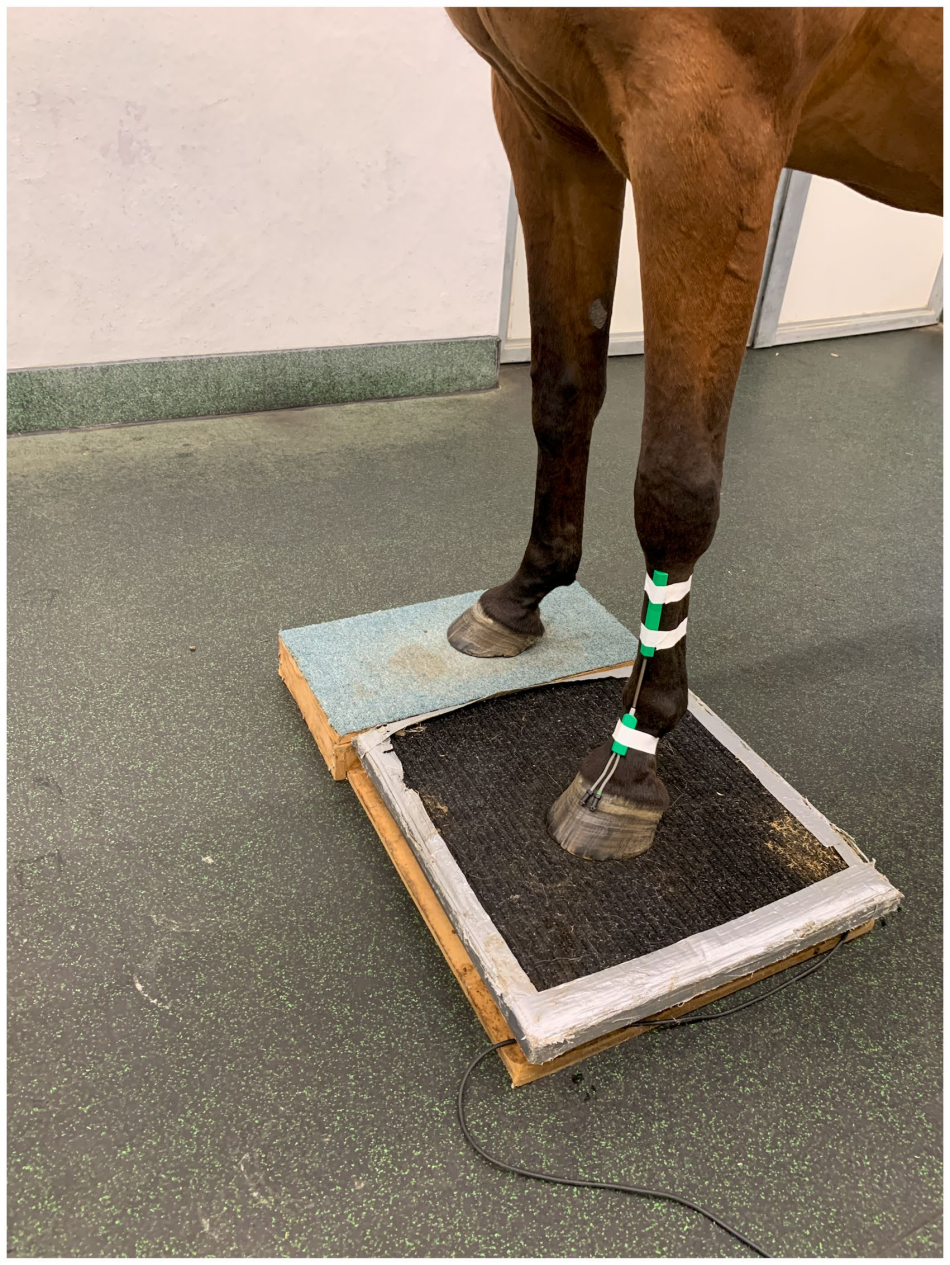
Twin-axis SG150B goniometer attached to the dorsum of a thoracic limb. The distal base plate extends to 1 cm proximal to the coronary band, with the proximal base plate extending to the metacarpal tuberosity. The two are connected by an extensible lead.

A twin-axis SG150B goniometer (Biometrics Ltd, Newport, United Kingdom) was secured to the dorsal aspect of the distal thoracic limb using zinc oxide tape (Figure 1). It was placed such that the distal (short) base plate lay on the dorsal pastern finishing 1 cm proximal to the coronary band, and the proximal (long) base plate was fixed to the dorsal aspect of the third metacarpal bone, with its most proximal extent at the level of the metacarpal tuberosity. The goniometer was connected to an Angle Display Unit (Biometrics Ltd, Newport, United Kingdom) via a 1.5-m interconnecting lead such that the angle of the MCP joint was displayed. A set of 600 kg × 0.2 kg floor scales (My Scales, Milton Keynes, United Kingdom), placed in a custom-made wooden box, was used to determine the mass and thus vertical GRF. The wooden box housed the floor scales such that one limb could bear weight on the scales and the contralateral limb on the box itself (Figure 1). Each limb had 5 measurements taken simultaneously of both angle and weight with the horse stood square, alternated with 5 measurements with the contralateral limb elevated. This generated 10 data points on a graph of weight plotted against the MCP joint angle for each limb. The gradient of a line of best fit was determined using Microsoft Excel (Microsoft Corporation, Redmond, WA, United States), and thus, a limb stiffness index for each limb was generated.

The accuracy of the goniometer to measure the angle of the metacarpophalangeal joint was assessed in one horse. Ten lateromedial radiographs were taken of the metacarpophalangeal joint with the goniometer positioned as in Figure 1. Five radiographs were taken with the limb weight-bearing, and five with the contralateral limb lifted, goniometer readings were recorded at the same timepoint as each radiograph. Metacarpophalangeal joint angle was measured digitally using Visbion image viewing software by a single user, by measuring the angle of intersection of a line parallel to the midshaft of the third metacarpal bone and a line parallel to the midshaft of the proximal phalanx.

The repeatability goniometric limb stiffness measurements (method 2) were evaluated using a single animal. Once the limb stiffness index for the limb was determined, the horse was moved off the scales, equipment was removed from the leg, and the measurement process was repeated by the same operator. This was performed 5 times for each leg, generating 5 stiffness indices for each leg. Repeatability was assessed by calculating the coefficient of variation (C_V_) for each limb. The lower the Cv, the smaller the dispersion of values around the mean, which indicates greater repeatability.

### Statistical analysis

Quantitative data were assessed for normality using the Shapiro–Wilk test and graphically. Normally distributed data were summarized using the mean (standard deviation [SD]). A mean limb stiffness index for both thoracic limbs of each horse was generated by each of the two methods, and these were plotted against one another in order to assess the correlation between methods. Pearson’s product moment correlation coefficient (r) was calculated for the datasets using Microsoft Excel to assess the strength and significance of correlation. Significant positive correlation coefficients were interpreted as weak (<0.35), moderate (0.35 to <0.67), high (0.67 to <0.90), and very high correlations (≥0.90) (30).

A Bland–Altman plot was also created for the two datasets to assess agreement between the methods using GraphPad Prism (GraphPad Software, 225 Franklin Street, Fl, Boston, MA). Two lines were used to represent the mean difference between the two methods and the limits of agreement to indicate whether there are systematic differences between the two methods. The limits of agreement were defined as the mean difference ± 1.96 SD of differences to indicate the range of differences. The mean was calculated for both the absolute difference and percentage difference between the left and right limbs, for method 1 and method 2. The absolute difference for each horse was then compared, between method 1 and method 2, with a paired t-test.

## Results

The mean kinetically derived limb stiffness (method 1) for the 6 horses included in the present study was 12.28 kg/degree (SD 3.22). The mean goniographically measured limb stiffness (method 2) for the same 6 horses was 9.25 kg/degree (SD 2.47).

There was close agreement between the metacarpophalangeal joint angle measured simultaneously with the goniometer and lateromedial radiographs ten times in one horse, with a mean difference of 1.5° (± 0.9°).

Results of repeatability testing performed on a single animal using goniometric determination of limb stiffness are shown in Table 1. The coefficient of variation (C_v_) was 5.70%, indicating a small dispersion of values around the mean and repeatability of limb stiffness measurement using the novel clinical tool.

**Table 1:** Results of repeatability measurements using goniometric determination of limb stiffness (method 2) of both thoracic limbs in a single horse, with each measurement repeated 5 times.

Limb	Test	Correlation coefficient (R)	Stiffness Index (kg/degree)	Mean (kg/degree)	Standard Deviation (kg/degree)
Left Fore	1	0.99	10.38	10.41	0.25
	2	0.99	10.46		
	3	1.00	10.77		
	4	1.00	10.08		
	5	0.99	10.36		
Right Fore	1	0.99	9.82	9.51	0.40
	2	1.00	9.22		
	3	0.99	9.03		
	4	0.99	9.50		
	5	0.99	10.00		

**Figure 2:**
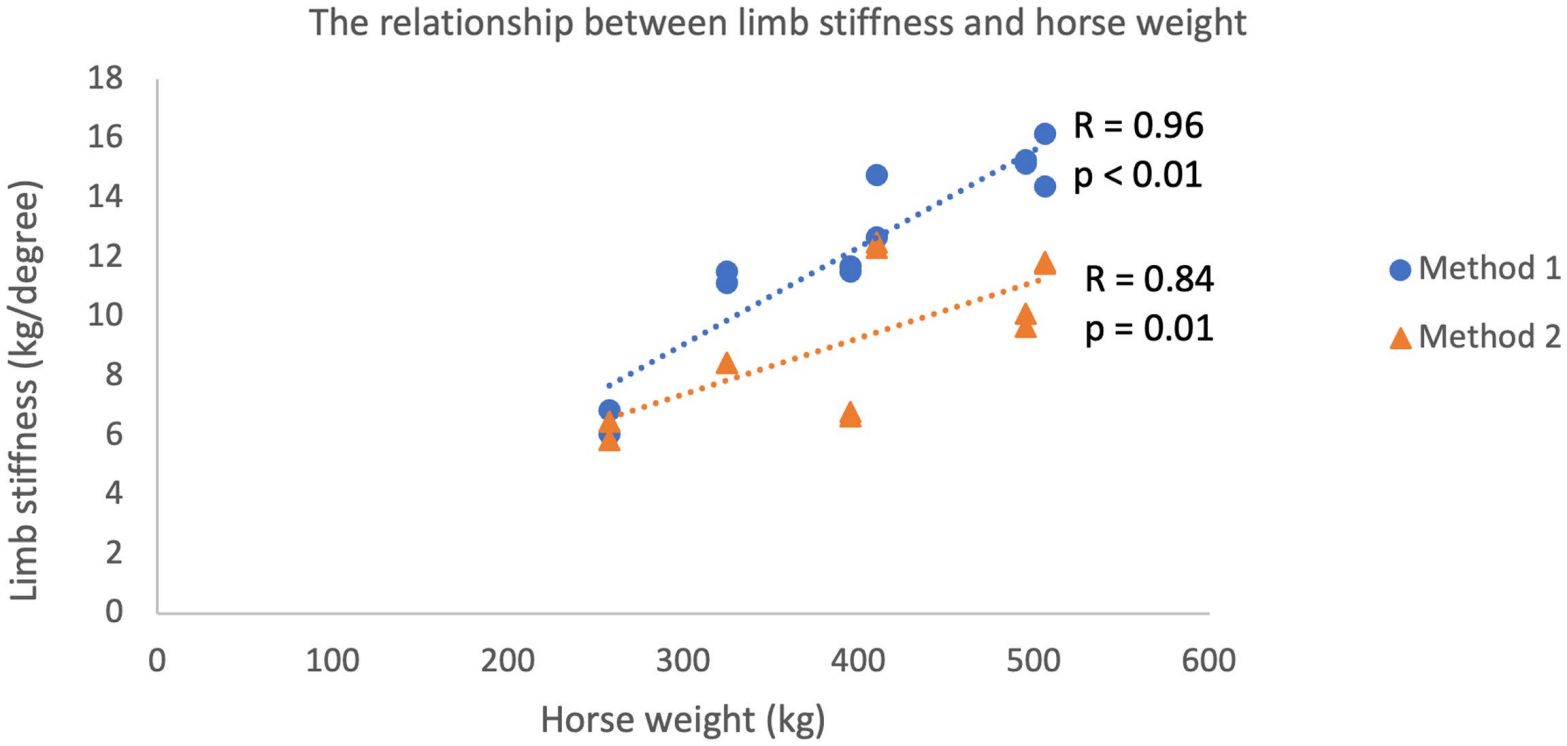
Linear relationship between limb stiffness as determined using both Method 1 (kinematic determination of limb stiffness) and Method 2 (goniometer and floor scales) and the mass of the subject. Both methods were performed in both forelimbs of 6 horses (total of 12 measurements).

**Figure 3:**
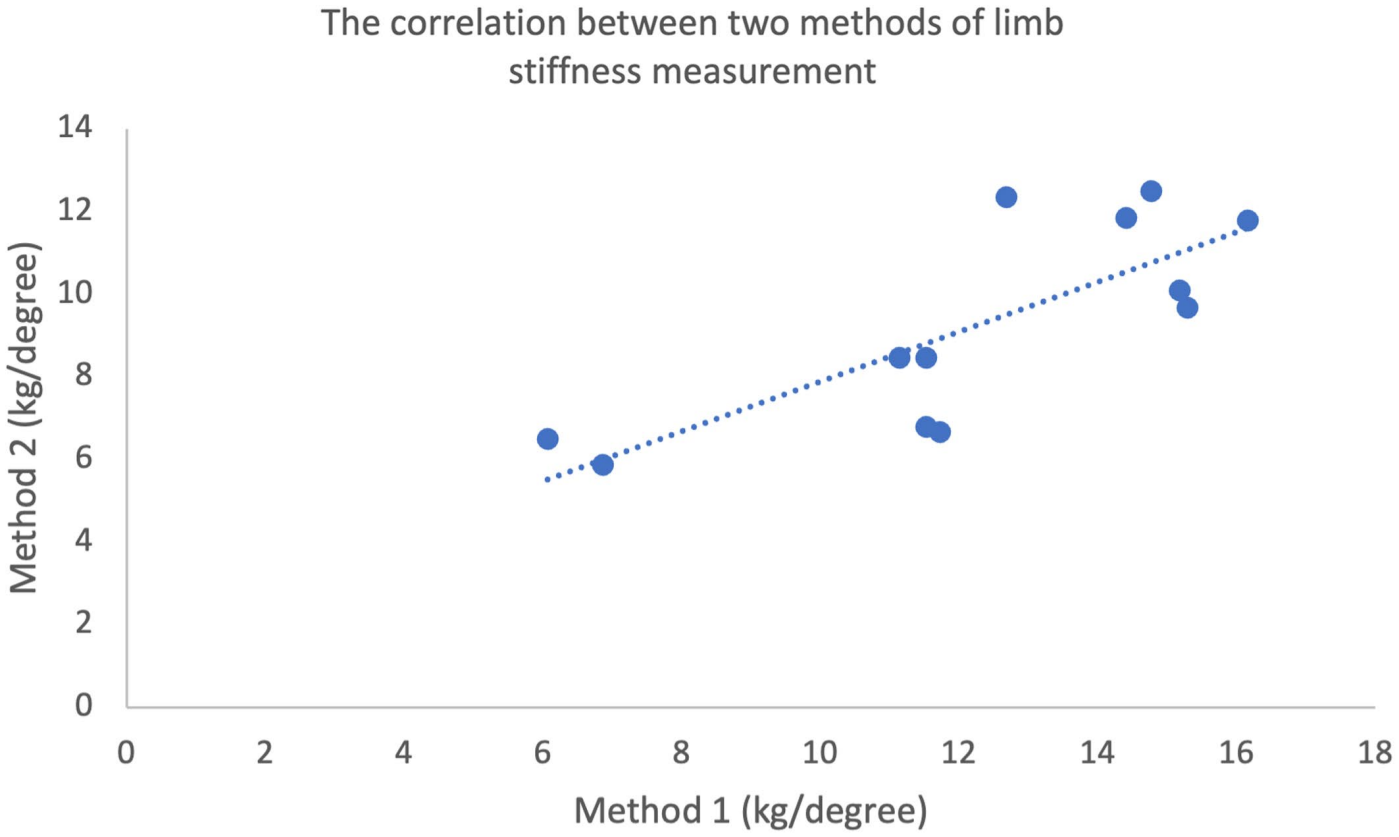
Correlation between the limb stiffness indices determined using Method 1 and Method 2 (*r* = 0.615, *p* < 0.01), measured in both forelimbs of 6 horses (total of 12 measurements).

Limb stiffness indices plotted against the mass of each subject generated for each method are shown in Figure 2. Pearson’s product moment correlation coefficient for the two datasets was calculated at *r* = 0.924 (*p* < 0.01) demonstrating a very high correlation between bodyweight and limb stiffness (30). Mean limb stiffness indices generated via the two methods are compared in Figure 3. Pearson’s product moment correlation coefficient for the two datasets was calculated at *r* = 0.784 (p < 0.01) indicating a high correlation between methods (30).

**Figure 4:**
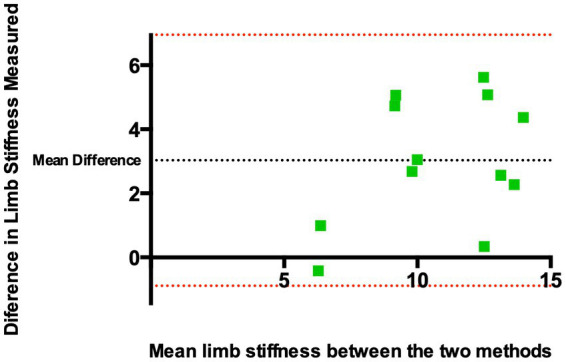
Bland–Altman plot of the difference of derived limb stiffness indices (kg/degree) between Method 1 and Method 2 against the mean limb stiffness from both methods. The black dashed horizontal line represents the mean difference between the two methods, indicating whether there are systematic differences between the two methods. Red dashed horizontal lines represent limits of agreement, defined as the mean difference ± 1.96 SD of differences, and indicate the range of differences. The mean difference was found to be 3.03 kg/degree between methods. The 95% limits of agreement indicating the range of differences were −0.89 to 6.95 kg/degree (±3.92 kg/degree around the mean).

Figure 4 shows a Bland–Altman plot for the two datasets. This revealed a mean difference in limb stiffness index of 3.03 kg/degree between methods demonstrating that the kinematic-derived measurements (method 1) provided measurements that were systematically higher than the goniometer-derived measurements (method 2). The 95% limits of agreement indicating the range of differences were-0.89 to 6.95 kg/degree (±3.92 kg/degree around the mean).

The absolute and percentage differences in limb stiffness index between the left and right forelimb for both method 1 and method 2 are shown in Table 2. They indicate that method 2 shows reduced variation than method 1 (the gold standard), but this was not found to be significant (*p* = 0.146).

**Table 2:** Mean and standard deviation (SD) of the absolute and percentage differences in limb stiffness index between the left and right forelimbs of 6 horses, measured with two methods.

Difference in limb stiffness between left and right limbs				
	Method 1		Method 2	
	Mean	SD	Mean	SD
Absolute difference (kg/degree)	0.30	1.24	0.16	0.31
Percentage difference (%)	7.45	6.23	3.04	3.74

## Discussion

This study provides evidence that the presented novel method using an electrogoniometer and floor scales can be used in a clinical setting to non-invasively measure distal forelimb stiffness. The accuracy of fetlock angle measurement using a goniometer taped to the front of the distal limb in one horse was very good, as was the repeatability of limb stiffness measurement and correlation between methods, which had a low coefficient of variation although one limitation to the study was the small sample size. We therefore propose that the use of the goniometer for assessment of limb stiffness is sufficiently reliable for clinical use, with a variation that was lower than the alterations in stiffness indices identified in clinical cases (18). The procedure was non-invasive, well tolerated, and safe for animals and their handlers. Despite the electrogoniometer being attached to the skin spanning across the metacarpophalangeal joint, the interconnecting wire between the plates is coiled to allow unhindered elongation well over what was required during the different degrees of metacarpophalangeal joint flexion. The mobility of the skin would affect both methods of measurement, and it is its alignment to the dorsal cortices of the metacarpus and phalanges, which is the most relevant and unaffected by skin movement induced by flexion and extension of the metacarpophalangeal joint. The high level of repeatability demonstrated with both methods is consistent with this conclusion and is supported by the study of Bergh et al. (24) who demonstrated “fair” to “excellent” inter-tester reliability when measuring equine joint range of motion with an electrogoniometer positioned on the lateral aspect of the joint. Certainly, method 2 does not appear to give more variable results as this method showed a lower mean difference (with reduced standard deviation) in limb stiffness between the left and right limbs of individual horses than the kinematic analysis, although this was not statistically significant. However, when used in clinical practice, it must be considered that the novel method (method 2) may underestimate the value of limb stiffness by a mean of 3 kg/degree. In addition to this not being statistically significant, such a difference was considered by the authors unlikely to change clinical interpretation because of the need to compare limbs or for repeated measurements over time due to the variation of limb stiffness with weight. It does, however, indicate that the same method is used for each animal. It should also be noted that the definite value is not equal between both methods, and the definite values from the two methods should not be compared when measuring limb stiffness in future study.

A particularly interesting finding was the linear relationship between limb stiffness and body mass seen with both methods, which accounts for much of the inter-individual variation. This finding is of biomechanical significance for maintaining optimal elastic efficiency of the SDFT across equines of different sizes, supporting the previously proposed concept that the SDFT acts as a spring system for energy storage (31). The linear relationship between animal weight and limb stiffness necessitates normalizing the limb stiffness to bodyweight to allow comparison between individuals or alternatively expressing the ratio in limb stiffness between limbs.

Potential applications for this proposed clinical tool are numerous. Dakin et al. (18) found limb stiffness to normalize by 7 months post-SDFT injury, but data beyond this point were not obtained. Therefore, significant changes after this could have implications both for reduced energy efficient locomotion and increased risk of re-injury. The ability to compare the mechanical properties of treated and control limbs could also be a key clinically relevant mechanical parameter when evaluating the efficacy of novel treatment options for SDFT tendinopathy (15, 32–35).

## Conclusion

The current study offers validation of a potentially useful clinical and research tool for the assessment of equine limb stiffness *in vivo.* The proposed technique showed a statistically significant relationship to the current ‘gold standard’ for measuring limb stiffness, had good repeatability, was safe to perform, and was well tolerated by subjects. Further study is required to collect data from a range of equines, including animals with SDF tendinopathy and other soft tissue injuries of the distal limb, to further evaluate the accuracy with respect to a clinically meaningful outcome in clinical cases.

## Data availability statement

The raw data supporting the conclusions of this article will be made available by the authors, without undue reservation.

## Ethics statement

The animal study was approved by the Royal Veterinary College Clinical Research Ethical Review Board. The study was conducted in accordance with the local legislation and institutional requirements.

## Author contributions

BJ: Writing – original draft. KH: Writing – review & editing. SG: Writing – original draft. AL: Writing – original draft. RT: Writing – original draft. AF-J: Writing – original draft. RS: Writing – original draft, Writing – review & editing.
